# Machine learning-based clinical mastitis detection in dairy cows using milk electrical conductivity and somatic cell count

**DOI:** 10.3389/fvets.2025.1671186

**Published:** 2025-11-14

**Authors:** Lihong Pan, Xiao Chen, Ding Han, Nan Li, Deyong Chen, Junbo Wang, Jian Chen, Xiaoye Huo

**Affiliations:** 1 State Key Laboratory of Transducer Technology, Aerospace Information Research Institute, Chinese Academy of Sciences, Beijing, China; 2 School of Advanced Interdisciplinary Sciences, University of Chinese Academy of Sciences, Beijing, China; 3 School of Future Technology, University of Chinese Academy of Sciences, Beijing, China; 4 School of Electronic, Electrical and Communication Engineering, University of Chinese Academy of Sciences, Beijing, China

**Keywords:** somatic cell count, electrical conductivity, mastitis detection, machine learning, neural network, dairy cows

## Abstract

Bovine mastitis, a prevalent disease causing substantial economic losses in dairy production, requires accurate and robust detection methods. Traditional threshold-based approaches using electrical conductivity (EC) are limited by low specificity and farm-specific variability. While somatic cell count (SCC) offers a more reliable biomarker for intramammary inflammation, current SCC sensors often yield imprecise data and are costly to implement, resulting in a lack of accurate, quantitative, and widely applicable models for mastitis monitoring. This study presents an machine learning-based diagnostic framework integrating logistic regression (LR), support vector machines (SVM), and feedforward neural networks (FNN) to evaluate mastitis detection performance with EC, SCC, and their combined inputs. Using data from 93 cows across four dairy farms, we demonstrate that SCC-based models consistently outperform EC-based approaches. The SVM model achieved 95.6% accuracy and 100% sensitivity when utilizing SCC as input feature. The FNN model attained the highest AUC (0.981), highlighting neural networks’ capability to capture complex patterns. Although the addition of EC to SCC did not improve performance across all metrics, it showed potential to enhance robustness in contexts where accurate SCC data are limited. These findings underscore the diagnostic superiority of SCC and the potential of tailored machine learning solutions in modern dairy production settings. Future work should focus on expanding datasets across multiple regions and integrating high-precision SCC sensors for real-time deployment in automated detection systems.

## 1 Introduction

Mastitis, an inflammation of the mammary gland typically caused by bacterial infection (1–3), is one of the most prevalent and economically devastating diseases in global dairy production (4, 5). Approximately 30–50% of the global dairy cows experience mastitis at some point in their lives, resulting in annual economic losses of up to $35 billion (6–9). Mastitis not only reduces milk production and significantly deteriorates milk quality through altered composition, but also severely compromises herd health. Therefore, developing reliable mastitis detection methods is crucial for ensuring the safety of dairy product, improving the welfare of dairy cows, and minimizing economic losses in dairy farming (10, 11).

Various diagnostic approaches have been developed to assess udder health, ranging from manual clinical examinations to quantitative methods integrated into routine milking processes. With increasing interest in precision dairy farming, there has been a growing focus on developing tools capable of enabling timely and accurate mastitis detection that support more efficient and consistent monitoring (12, 13). These systems typically rely on various milk-related detection indicators, such as pH (14), electrical conductivity (EC) (6), color (15), somatic cell count (SCC) (16), milk yield (17), clotting characteristics (18), and cow behavior (19).

Among these indicators, EC remains the most widely implemented indicator in commercial mastitis detection systems, including prominent platforms such as DeLaval (DeLaval International AB, Sweden) and Lely (Lely Industries N.V., Netherlands). Elevated EC values are generally associated with mastitis, and diagnostic decisions are often made by comparing measured EC values to predefined thresholds (20). However, despite its widespread adoption, there is considerable inconsistency in the reported EC thresholds. For example, Lazaro et al. (21) reported that milk conductivity is typically below 4.9 mS/cm in healthy cows, 4.9–5.15 mS/cm in subclinical cases, and above 5.15 mS/cm in clinical mastitis, whereas Yesil et al. (22) reported a different broader range, suggesting that mastitis is suspected when the EC value exceeds an average threshold of 5–5.5 mS/cm. This lack of standardization reflects the fact that EC is influenced by a variety of non-infectious factors, which compromises its diagnostic reliability. Consequently, the sensitivity and specificity of EC-based mastitis detection remain low. For instance, Bausewein’s cross-sectional study (23) revealed that mastitis detection in DeLaval and Lely milking systems, when based on EC measurements alone, achieved only 61–78% sensitivity, highlighting the limitations of relying solely on this parameter.

In contrast to EC, SCC provides a more direct and reliable indication of udder health, reflecting the inflammatory response through increased leukocyte counts in milk (24–26). As a result, SCC is widely regarded as a key biomarker for mastitis detection. To ensure the quality and safety of dairy products, many countries have established regulatory limits for SCC in milk. At the same time, some researchers are also actively promoting the use of accurate SCC thresholds as diagnostic criteria for mastitis (22, 27). However, the thresholds for SCC vary significantly across different regions (28–30), reflecting discrepancies in mastitis management standards.

While SCC is a well-established indicator of mastitis, values obtained from automated in-line sensors often exhibit significant inherent variability, compromising their diagnostic reliability in practice. Consequently, although numerous studies have attempted to model the relationship between SCC and mastitis at both the individual cow (25) and herd level (31) using such online data, the performance of these models remains suboptimal. For instance, Nagy et al. (32) reported that a model based on online SCC data achieved only 54.0% sensitivity and 77.0% specificity. This fundamental issue of data inaccuracy inevitably constrains the performance of any diagnostic algorithm built upon it, leading to widely disparate and often unsatisfactory results across studies and hindering the development of robust, generalizable solutions. Building on this need for more robust and adaptable diagnostic strategies, machine learning (ML) techniques have been increasingly adopted in mastitis detection to overcome the limitations of threshold-based methods (33–35). Unlike traditional approaches that rely on fixed threshold values for single indicators, ML models can integrate heterogeneous features and learn nonlinear relationships between input variables and mastitis status. This multi-parameter capability enhances their adaptability and predictive accuracy across varying herd conditions and sensor performance (36–40). For instance, Tian et al. (14) implemented a k-nearest neighbor (KNN) model with EC and pH inputs. Similarly, Khan et al. (19) developed a support vector machine (SVM) classifier based on cow behavior data, while Bobbo et al. (17) achieved an accuracy of 79.7% and sensitivity of 52.4% using a linear discriminant analysis (LDA) for mastitis diagnosis. Although model performance varied, these studies demonstrate that combining multiple indicators through ML-based methods offers a promising route toward more robust and accurate mastitis detection compared to single-threshold systems.

In summary, although EC and SCC are widely used for mastitis detection (41), their diagnostic reliability is limited by inconsistent thresholds, inherent sensor inaccuracies, and farm variability. At the same time, farmers face increasing demands for more accurate and reliable mastitis detection, as current threshold-based methods often lead to delayed interventions, higher treatment costs, and reduced milk quality. Machine learning offers the potential to transform mastitis management by integrating multiple parameters into flexible, data-driven models that improve diagnostic accuracy and provide farm-adaptable solutions. Thus, evaluating and comparing machine learning algorithms using accurate and reliable EC and SCC data remains a research need with substantial potential to advance mastitis management practices.

## 2 Materials and methods

### 2.1 Working principle

**Figure 1:**
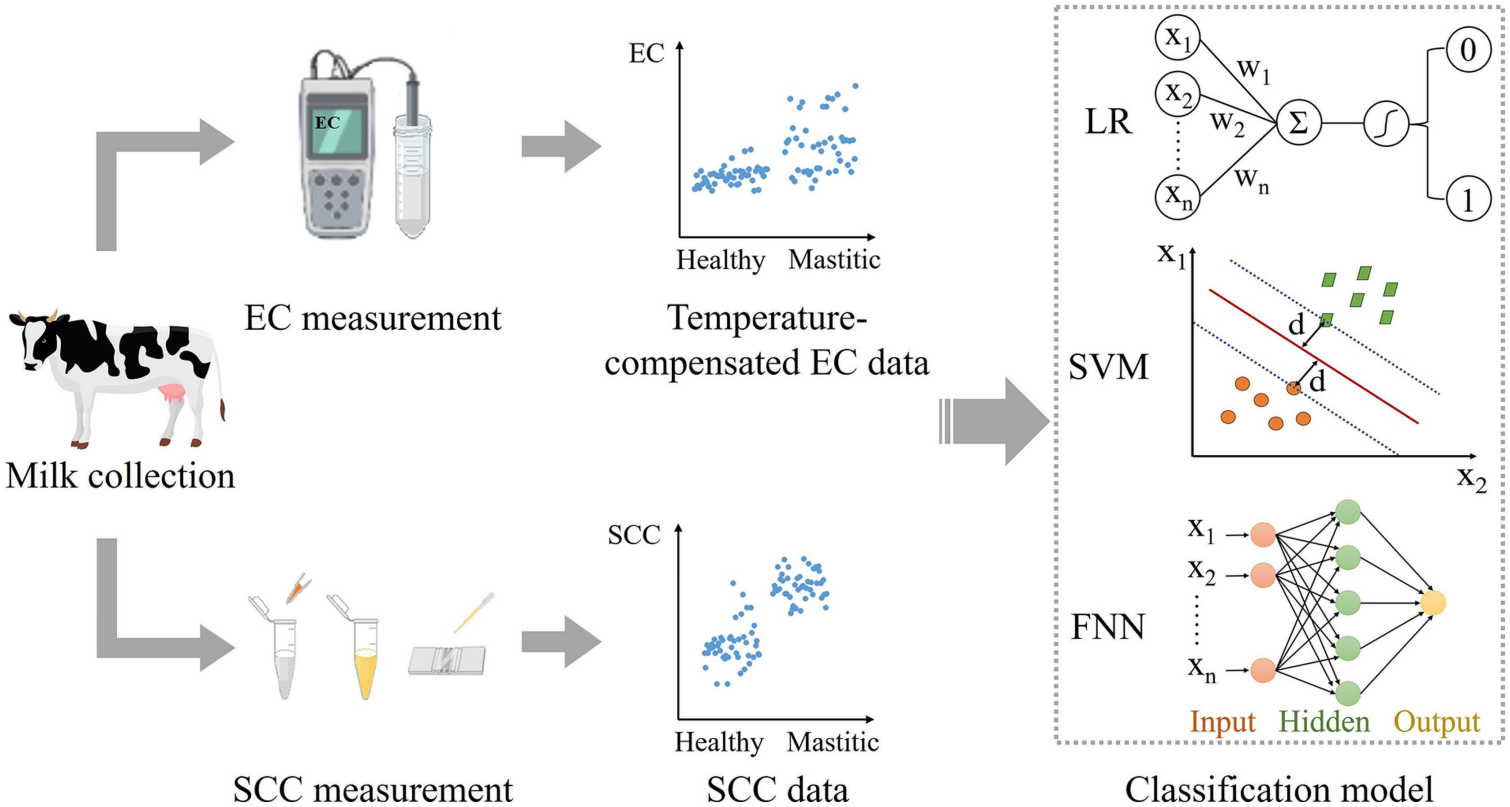
Workflow for mastitis diagnosis using AI classification models. The process begins with raw milk sample collection from dairy farms, followed by measurement of electrical conductivity (with temperature compensation) and somatic cell count. After data preprocessing, the processed features serve as input for binary classification models to predict mastitis status.

As shown in Figure 1, the study took a systematic approach designed to ensure robustness across diverse dairy farms and environmental conditions. The workflow begins with the collection of raw milk samples from dairy farms. Following acquisition, each sample underwent dual-parameter measurement, including EC and SCC. The EC was measured with temperature compensation to account for environmental variability. The SCC was determined through fluorescence microscopy. The curated data served as input for two machine learning models (LR, SVM) and one neural network (FNN), with performance validation followed.

### 2.2 Sample collection

From January to December 2024, milk samples were collected from cows that were randomly selected from four dairy farms in Beijing. The study consisted of 47 healthy cows and 46 cows with mastitis, as determined by veterinary clinical diagnosis with the signs of inflammation in the udder. To ensure sample independence, all samples were collected from different cows. Cows within the first 4–5 days postpartum and those in the late lactation stage were excluded from sampling, as physiological elevations in SCC commonly occur during these periods and are unrelated to mastitis. During each milking session, the first three streams of milk were discarded, and the subsequent 30–50 mL of milk was collected for analysis.

### 2.3 EC measurement

The electrical conductivity of each milk sample was measured using a conductivity meter (HANNA HI8733 Multi-range EC Meter, Italy). Prior to each measurement, the electrode tip was thoroughly cleaned to prevent contamination and ensure accuracy. Each sample was measured three times, and the average value was used to enhance measurement reliability and consistency. Given the strong influence of temperature on conductivity, temperature compensation was applied during each measurement to eliminate temperature-induced variation.

### 2.4 SCC measurement

To ensure the accuracy of the dataset used for model development, microscopic examination for somatic cell counting was employed in this study. To reduce the cell loss associated with centrifugation and washing, direct fluorescence staining was performed on raw milk samples without a washing step. Fluorescent dyes were used instead of traditional stains such as trypan blue to enhance image contrast in low-transparency milk. Specifically, 100 μL of fresh raw milk was mixed with 2 μL of 1 mg/mL acridine orange staining solution (Thermo Fisher Scientific, USA). The stained samples were then applied to cell counting slides (Bio-Rad Laboratories, USA). Observations were performed using a fluorescence microscope (Olympus IX73, Japan) equipped with a 10 × objective lens, and images were captured using a Prime BSI Express sCMOS camera (USA). For each sample, multiple random fields of view were selected for counting, and the average value was calculated to enhance accuracy and reliability. For samples with extremely high somatic cell counts, dilution was performed accordingly.

### 2.5 Data processing and algorithms

The collected EC and SCC data were normalized using Z-score standardization, where each value was transformed by subtracting the mean and dividing by the standard deviation. This eliminated dimensional differences and facilitated subsequent analysis. Cow health status was numerically encoded, with healthy cows labeled as 0 and mastitic cows as 1, enabling binary classification.

All data processing, model construction, and result visualization of three classification algorithms were performed using MATLAB (The MathWorks, USA). The three models were selected for this study based on their complementary characteristics. LR was chosen for its interpretability and efficiency, SVM for its robustness in high-dimensional spaces, and FNN for its ability to model complex nonlinear relationships. Furthermore, the models span a range of complexity, from simple to advanced, making them effective representatives of machine learning and neural network to evaluate and compare their mastitis detection performance.

EC, SCC, and their combination were used as input features. To make full use of the limited data and enhance generalization, five-fold cross-validation was applied instead of a single train–test split. The data were shuffled before training to eliminate order bias and improve model stability. Hyperparameter tuning was conducted for LR, SVM, and FNN to optimize performance and obtain the best configurations. For the LR, we adjusted the regularization strength (Lambda, 1–1,024) and classification threshold (0–1); for the SVM, we optimized the kernel function (linear/RBF/polynomial), kernel scale (0.001–10), and box constraint (1–1,024); and for the FNN, we varied the hidden layer size (5–50 neurons), learning rate (0.01–0.2), and regularization (0–0.5). The best model for each classifier was selected based on the highest test accuracy, with AUC as a secondary criterion.

### 2.6 Evaluation of the algorithms

In this study, the performance of each classifier was comprehensively evaluated using various feature inputs on the test dataset. Receiver operating characteristic (ROC) curves were generated for all combinations of classifiers and input features to visually assess the trade-off between true positive rate (sensitivity) and false positive rate (1—specificity) across different thresholds, thereby illustrating the classifier’s discriminatory power. Quantitative evaluation was further conducted using accuracy, sensitivity, specificity, and the area under the curve (AUC). Accuracy was defined as the proportion of correctly classified instances out of the total number of instances, providing an overall effectiveness measure of the classifier. Sensitivity, also known as the true positive rate, was measured to evaluate the classifier’s ability to correctly identify positive instances. Sensitivity is particularly important in scenarios where accurately identifying positive cases is critical. Specificity, or true negative rate, was assessed to determine the classifier’s ability to correctly identify negative instances. Specificity is important where false positives are costly or undesirable. These metrics were expressed as:


$$A\text{ccuracy}=\frac{\mathrm{TP}+\mathrm{TN}}{\mathrm{TP}+\mathrm{TN}+\mathrm{FP}+\mathrm{FN}}$$

$$\text{Sensitivity}=\frac{\mathrm{TP}}{\mathrm{TP}+\mathrm{FN}}$$

$$\text{Specificity}=\frac{\mathrm{TN}}{\mathrm{TN}+\mathrm{FP}}$$

where TP, TN, FP, and FN represent true positives, true negatives, false positives, and false negatives, respectively.

AUC, derived from the ROC curve, provides a scalar summary of classifier performance across all thresholds. A value of 1 indicates perfect classification, while 0.5 suggests random guessing. AUC is particularly informative for comparing classifiers and evaluating models under class imbalance. Together, these metrics enabled a robust evaluation of each classifier across different feature sets, supporting optimal strategy selection for mastitis detection.

## 3 Results and discussions

**Figure 2:**
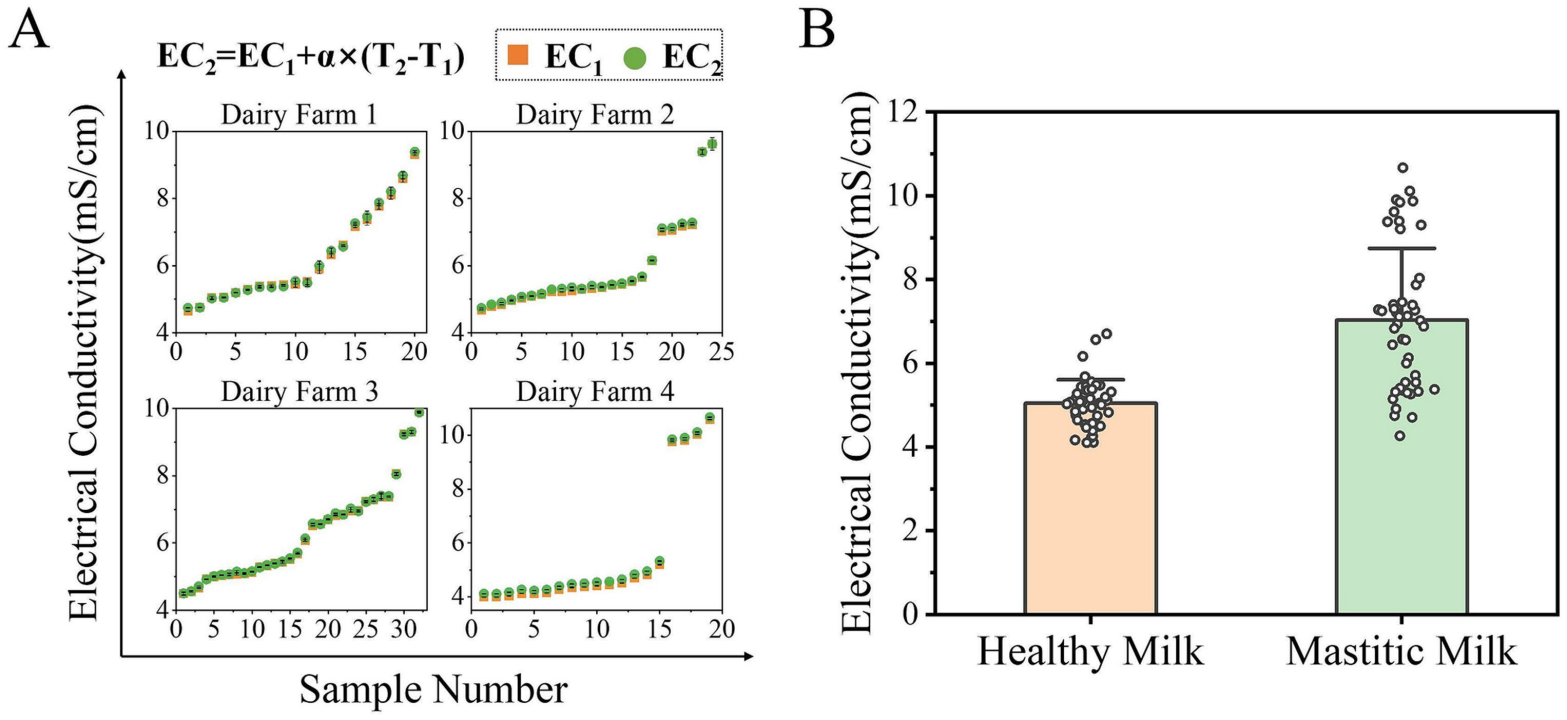
Temperature compensation and comparative analysis of electrical conductivity data. **(A)** Raw EC data from four dairy farms were temperature-compensated to 25 °C using a temperature coefficient (*α*) of 2.83% °C^−1^. EC_1_ and EC_2_ represent the original and temperature-compensated conductivity values, respectively. **(B)** Distribution of temperature-compensated EC values for healthy and mastitic milk. While mastitic milk exhibited higher average conductivity compared to healthy milk, substantial overlap between distributions limits the reliability of EC as a standalone diagnostic indicator for mastitis.

In this study, the performance of multiple ML models was evaluated by using different feature input configurations, including EC alone, SCC alone, and their combination. Figure 2 illustrates the comprehensive analysis of EC data of milk across four dairy farms. To eliminate interference of temperature on EC, the original EC data of each dairy farm were temperature-compensated as shown in Figure 2A. The compensation was performed using the linear model: EC_2_ = EC_1_ + *α* (T_2_ – T_1_), where EC_1_ represents the original measured conductivity, EC_2_ is the temperature-compensated value (standardized to 25 °C), α is the temperature coefficient, and T_1_ and T_2_ denote the sample and reference temperatures, respectively. The temperature coefficient (*α*) was determined to be 2.83% °C^−1^. This value was derived from a calibration experiment, wherein a raw milk sample was subjected to controlled temperature variations across a range from 15 °C to 40 °C using a water bath. The electrical conductivity was measured in triplicate. A linear regression model was then fitted to the dataset, and the coefficient from this regression was adopted as α. While this coefficient was determined using a single milk sample, we acknowledge that inter-sample and inter-farm variations in milk composition could influence this coefficient, and future work should aim to establish a more generalized coefficient based on a larger and more diverse set of samples.

The EC values revealed distinct distributions between healthy milk and mastitic milk, as shown in Figure 2B. The results showed that the average conductivity of the mastitic milk was 7.03 mS/cm, which was significantly higher than that of healthy cows (5.04 mS/cm). However, although conductivity serves as a valuable indicator for mastitis, it manifests substantial limitations. The conductivity distributions of healthy and mastitic milk exhibits significant overlap, compromising its reliability as a standalone indicator for mastitis diagnosis. In practice, no clear threshold can reliably separate healthy and infected cases across diverse farm conditions. Moreover, milk conductivity demonstrates notable farm-to-farm variability and is influenced by multiple physiological and management factors unrelated to mastitis, such as milking frequency, dietary composition, and water consumption patterns. These findings highlight both the potential value and practical limitations of EC in mastitis detection, supporting the need for its integration with other indicators and advanced data-processing methods, such as machine learning, to enhance diagnostic accuracy.

**Figure 3:**
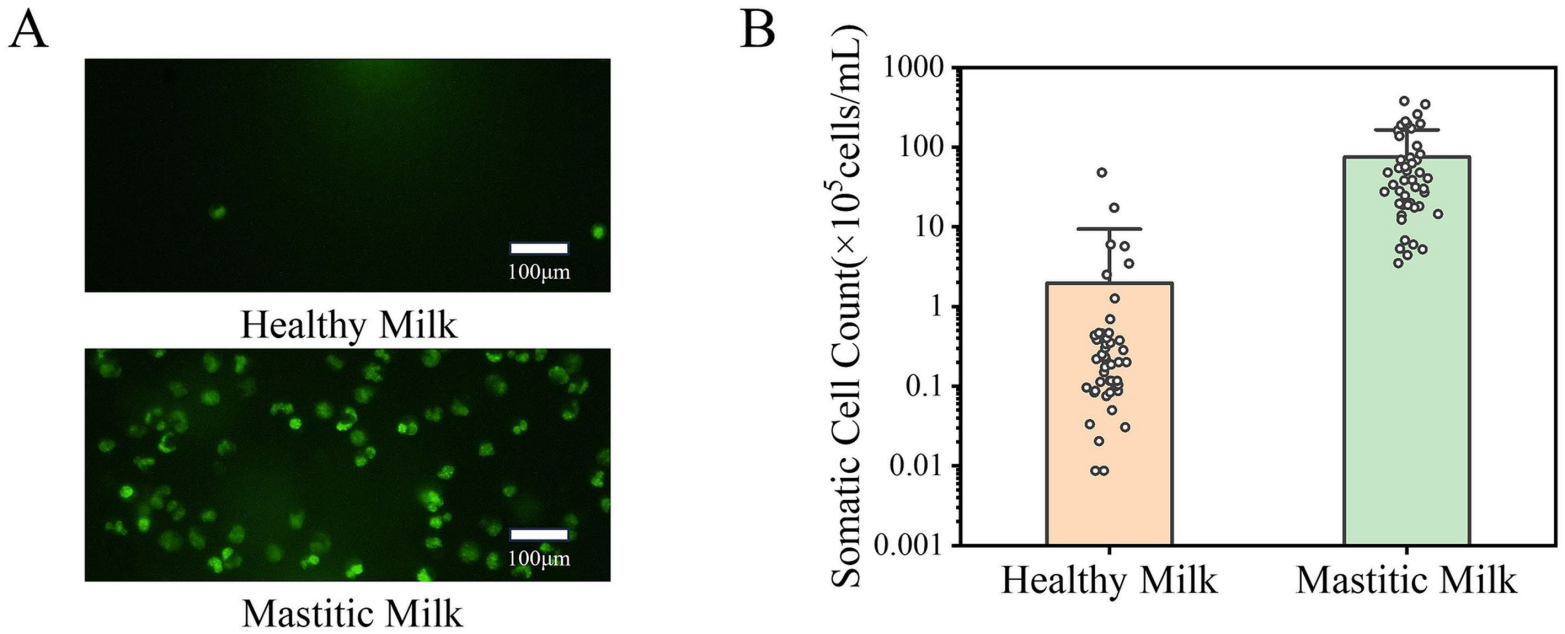
Diagnostic value of somatic cell count for mastitis detection. **(A)** Fluorescent microscope images of somatic cells in healthy milk (upper) and mastitic milk (lower), stained with a nucleic acid-specific fluorescent dye. **(B)** Distribution of SCC data for healthy and mastitic milk, with less overlap compared to EC data. Statistical analysis confirmed that SCC as a more reliable and specific indicator of mastitis.

Figure 3 illustrates the diagnostic characteristics of SCC, which demonstrated superior diagnostic performance compared to EC in distinguishing mastitic from healthy milk. Fluorescent microscope images of somatic cells in healthy milk and mastitic milk are shown in Figure 3A. Since a nucleic acid fluorescent dye staining both DNA and RNA was used, all somatic cells including lymphocytes, polymorphonuclear neutrophils (PMNs), macrophages and other cell types were effectively stained. The fluorescence microscope images clearly demonstrate a significantly higher somatic cell count in mastitic milk compared to healthy milk.

This visual distinction was validated in quantitative analysis, with Figure 3B showing the distribution of SCC data for healthy and mastitic milk, and SCC values spanning four orders of magnitude (10^4^–10^7^ cells/mL). Notably, the overlap in SCC data between healthy milk and mastitic milk is less pronounced than that in EC data, with only 4.3% of samples fell within the overlap range, a marked improvement over the 18.3% overlap of EC data. The results indicate that the classification method based on SCC can better distinguish between healthy and mastitic milk, and SCC was a more reliable and specific indicator of mastitis compared to EC.

**Figure 4:**
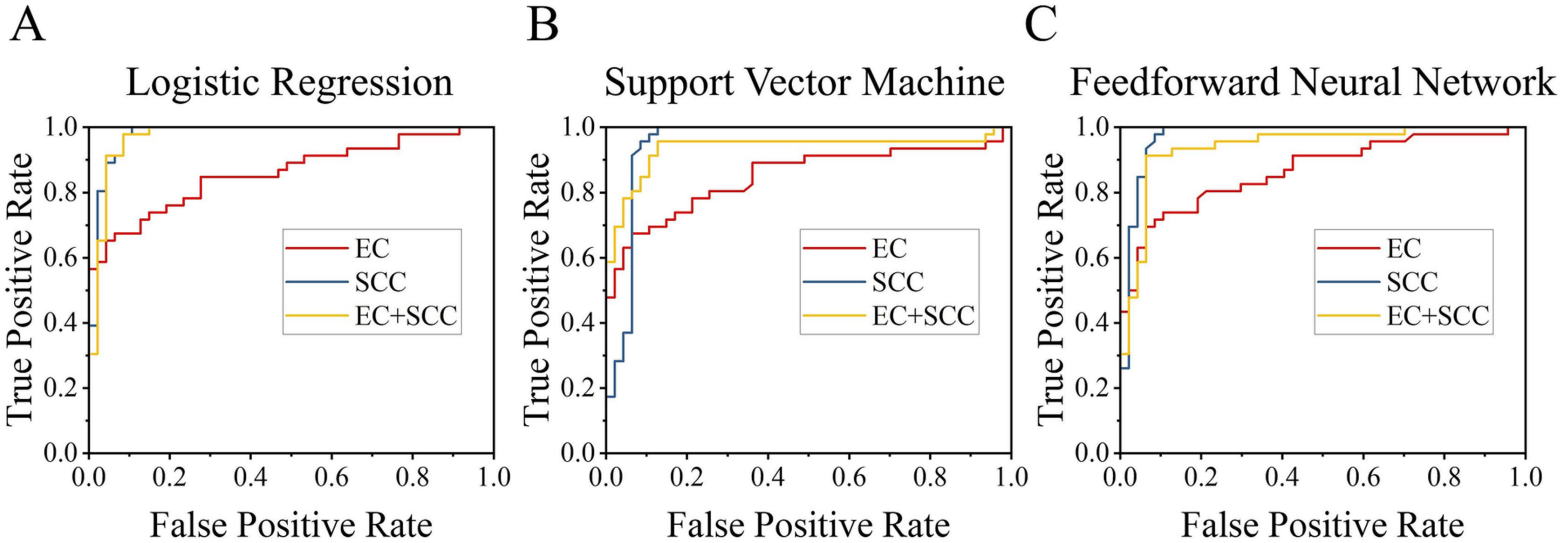
ROC curves comparing diagnostic performance of different models using distinct input features (EC, SCC and their combination). **(A)** Logistic regression model. **(B)** Support vector machine model. **(C)** Feedforward neural network model.

With the EC and SCC data from different samples, binary classifications were performed using various classification models and different input features, including EC, SCC and their combination. The diagnostic performance of three classifiers (LR, SVM, FNN) was then characterized thoroughly. As shown in Figure 4, the ROC analysis provides a visual assessment of each model’s ability to distinguish between healthy and mastitic milk at different thresholds. Figures 4A–C show the ROC curves for LR, SVM, and FNN models, respectively.

Different models exhibit varying performance and sensitivity-specificity trade-offs, suggesting that it is particularly important for farmers to choose the right model for their needs—whether to prioritize high sensitivity for early detection of mastitis, or high specificity to avoid false alarms. Notably, all models consistently showed higher AUC with SCC input compared to EC alone, further validating the reliability of SCC as a primary indicator in mastitis detection.

The performance metrics of different models (LR, SVM, FNN) and input features (EC, SCC, EC + SCC) were systematically analyzed and presented in Table 1. For comparison, performance indicators for classification using the SCC thresholds specified in the American National Standards (30) and recently reported models from literature (14, 17, 19, 32) are presented in the table. The results demonstrated that models utilizing SCC consistently outperformed those relying solely on EC. Interestingly, when SCC was used as an input feature, the addition of EC input feature did not improve the classification performance as expected, but instead, it led to slight decreases in most metrics, as shown in Table 1. This may be attributed to the variability and weaker discriminative power of EC, which introduced noise rather than complementary information to the SCC-based classification.

**Table 1:** Performance evaluation and comparison of different models.

Model	Input	Accuracy	Sensitivity	Specificity	AUC
LR	EC	0.797	0.653	0.936	0.850
	SCC	0.946	0.978	0.913	0.976
	EC + SCC	0.936	0.978	0.896	0.973
SVM	EC	0.797	0.631	0.960	0.843
	SCC	0.956	1.000	0.913	0.952
	EC + SCC	0.925	0.933	0.918	0.941
FNN	EC	0.806	0.673	0.936	0.865
	SCC	0.947	0.978	0.916	0.981
	EC + SCC	0.925	0.911	0.936	0.941
Threshold = 750,000 cells/mL [30]	SCC	0.914	0.870	0.957	–
kNN [14]	EC, pH, etc.	0.946	0.870	–	–
LDA [17]	Milk yield, composition, etc.	0.797	0.524	0.909	–
SVM [19]	Cow behavior	0.892	0.878	0.901	–
FNN [32]	SCC	–	0.540	0.770	–

Among all evaluated models, SVM model performed best in terms of accuracy and sensitivity with the input of SCC feature, with an accuracy of 95.6% and a perfect sensitivity of 100%. The FNN with SCC input showed the highest AUC value (98.1%) by effectively capturing non-linear relationships in the data, indicating that the comprehensive performance of the classifier is better. These metrics not only surpassed those using the current SCC threshold of the US national standard, but also exceeded the results reported in other relevant literature for comparable models in recent years. Notably, utilizing SCC values from online sensors (32) resulted in a 54.0% sensitivity and 77.0% specificity for diagnosing mastitis. In contrast, by employing high-accuracy SCC measurements and optimized ML models, our approach achieved better performance with 97.8% sensitivity and 91.6% specificity. These results suggest that with accurate SCC data, ML-based models have the potential to deliver significantly improved diagnostic outcomes compared to currently deployed methods.

The results demonstrate distinct performance characteristics between SCC and EC as mastitis indicators, with important implications for practical implementation. SCC consistently outperformed EC in classification accuracy and diagnostic reliability across all tested models. This superiority stems from SCC’s direct correlation with mammary gland inflammation, compared to EC’s susceptibility to non-infectious factors. Particularly noteworthy was the SVM model’s perfect sensitivity (100%) with SCC input, making it ideal for detection, albeit with increased false positives that may necessitate additional confirmatory tests. In contrast, the FNN model showed more balanced performance (AUC = 0.96), suggesting its suitability for operations where false alarms carry significant economic consequences. These findings highlight that optimal model selection should consider farm-specific factors including herd size, mastitis prevalence, and available resources for follow-up testing.

While SCC alone demonstrated superior diagnostic performance in this study, it is important to note that real-time, high-precision SCC measurement remains a technical challenge in many on-farm settings. In such cases, combining SCC with EC may enhance the model’s robustness by introducing complementary physiological information, particularly when SCC measurements are noisy, delayed, or intermittently unavailable. Notably, the combined input of EC and SCC yielded improved specificity in some models (e.g., SVM and FNN), which could be advantageous in reducing false positives in practical deployments. Therefore, multi-indicator fusion remains a valuable strategy, especially for scenarios where sensor limitations preclude consistent access to accurate SCC data.

The findings of this study underscore the necessity for advanced sensor technologies capable of accurately quantifying SCC. Although SCC measurements in this study were obtained under laboratory conditions, the findings provide valuable insights and benchmark references for the integration of accurate SCC data into automated detection systems. This work contributes to the ongoing development of reliable, AI-assisted diagnostic tools that can be embedded in future sensor platforms to improve mastitis management on modern dairy farms.

Moreover, the study demonstrates the adaptability of machine learning models, which can be tailored to various farm conditions and requirements to provide customized mastitis detection solutions. The classification of mastitis cases should be based on temporal urgency for intervention, rather than solely on their comparability to traditional non-sensor indicators of udder health.

However, it must be acknowledged that this study is an exploratory investigation with several limitations. First, the sample size was relatively small, with cows collected from a limited number of farms and from a single Holstein breed, which restricts the diversity of the dataset. Second, the classification relied solely on single timepoint samples based on veterinary clinical diagnosis, resulting in a cross-sectional dataset that does not capture the dynamic progression of the disease. These factors constrain the generalization capability of the models. Future research is recommended to utilize more extensive datasets collected under field conditions to comprehensively assess the model’s performance and practical applicability.

In light of these observations, the development of an innovative sensor-based mastitis detection and management system appears both necessary and promising. Currently, our ongoing work focuses on the development of high-precision SCC sensor technologies, and the optimization of machine learning models for integration into future automated diagnostic workflows in the context of smart agriculture. This dual approach aims to further reduce subjective misdiagnosis while improving the detection accuracy and robustness of mastitis in dairy cows, ultimately enabling more proactive herd health management.

## 4 Conclusion

In conclusion, this study shows that the use of accurate and reliable time-specific SCC or EC data enables machine learning algorithms to effectively classify milk samples into healthy and clinical mastitis categories. Furthermore, by systematically comparing LR, SVM, and FNN using EC, SCC, and their combination as inputs, we found that models based on SCC consistently outperformed those relying on EC. The SVM model achieved the highest accuracy (95.6%) and sensitivity (100%) with SCC as the primary input, while the FNN model delivered the best overall performance with an AUC of 0.981, highlighting its ability to capture complex patterns. These results underscore the value of SCC as a more reliable and specific indicator of mastitis, being less affected by non-infectious factors than EC.

Furthermore, although SCC alone yielded the best overall performance in this study, combining it with EC may still offer practical advantages in real-world applications where accurate real-time SCC acquisition is challenging. The incorporation of EC can enhance model robustness and diagnostic stability, particularly in settings lacking high-precision SCC sensors or where SCC measurements are intermittently unreliable.

While these ML models show great promise, their practical application requires further development in two key areas. First, large-scale validation across diverse herds and geographical regions will be essential to verify model robustness. Second, the development of high-precision SCC sensors integrated with edge computing-optimized algorithms could enable widespread deployment in automated detection systems. Such technological advancements would facilitate timely interventions, improve herd health management, and ultimately enhance milk quality and farm profitability.

## Data Availability

The original contributions presented in the study are included in the article/supplementary material, further inquiries can be directed to the corresponding author/s.
